# Compliance and Satisfaction With a Protocol for Identifying Novel Targets to Support Postpartum Opioid Use Disorder Recovery: Prospective Cohort Study

**DOI:** 10.2196/77899

**Published:** 2025-11-20

**Authors:** Alicia M Allen, Linnea B Linde-Krieger, Jendar Deschenes, Stephanie Mallahan, Alexandra Harris, Mariana Felix, Arushi Chalke, Alma Anderson, Priyanka Sharma, Katherine M King, Maddy T Grant, James Baurley, Lela Rankin, Stacey Tecot

**Affiliations:** 1 University of Arkansas for Medical Sciences, 4301 West Markham Street, Little Rock, AR, 72205, United States, 1 5013647377; 2 Arkansas Children’s Research Institute, Little Rock, AR, United States; 3 Department of Family and Community Medicine, University of Arizona, Tucson, AZ, United States; 4 School of Social Work Tucson, Arizona State University, Tucson, AZ, United States; 5 Clinical Translational Sciences, University of Arizona, Tucson, AZ, United States; 6 Epidemiology and Biostatistics, University of Arizona, Tucson, AZ, United States; 7 Laboratory for the Evolutionary Endocrinology of Primates, School of Anthropology, University of Arizona, Tucson, AZ, United States; 8 New York Consortium in Evolutionary Primatology, The Graduate Center, CUNY, New York, NY, United States; 9 Biorealm LLC, Monument, CO, United States

**Keywords:** cohort study, ecological momentary assessment, hormones, opioid use disorder, postpartum period, pregnancy

## Abstract

**Background:**

Although treatment for opioid use disorder (OUD) often yields high adherence during pregnancy, the risk of returning to opioid misuse during postpartum is high. There are currently no relapse prevention programs tailored to this unique time period. Using a prospective cohort study, we seek to preliminarily identify hormones or infant caregiving approaches as novel predictors of postpartum opioid misuse.

**Objective:**

As a first step in dissemination of results, this report contains a detailed account of the protocol, as well as recruitment, retention, compliance, and participant satisfaction.

**Methods:**

Participants were individuals with OUD (OUD+) and those without (OUD–) who were followed from late pregnancy (≥36 gestational wk) to postpartum month 5. From childbirth to postpartum week 12, participants completed daily surveys (capturing use, craving, interactions with infant) and weekly face-to-face visits (including collection of biological samples for hormone assays). Follow-up visits using the same procedures occurred at postpartum month 4 and 5.

**Results:**

Most participants (50 OUD+, 20 OUD–) notified the study staff of childbirth (n=63, 93%), completed at least 1 postpartum clinic visit (n=62, 87%), and completed follow-up (n=51, 73%). Compliance with procedures ranged from 81% for weekly surveys to 63% for weekly dried blood spots, generally with lower compliance among OUD+ and at later time points. Among a subgroup of participants (n=31), regardless of group and time point, reported high study satisfaction (eg, on a scale where 0 is “not at all” and 3 is “extremely,” on average, participants reported 2.9±0.4 for their willingness to complete this study again at week 12 postpartum).

**Conclusions:**

This prospective cohort study was well tolerated despite the challenging postpartum period. Data collected will provide ample opportunities to identify novel risk or protective factors to inform the development of new relapse prevention intervention programs specific to the needs of those with OUD during early postpartum.

## Introduction

Perinatal opioid use disorder (OUD) is increasingly common with most recent estimates indicating that up to 324 per 10,000 births are impacted [1-3]. Medication for OUD (MOUD) is the gold standard treatment, improving both maternal and infant outcomes [4-6]. However, the risk for return to opioid misuse and overdose increases during postpartum [7,8]. In fact, opioid overdose is now a leading cause of maternal mortality [9], with additional significant adverse impacts on the birthing person, infant, family, and beyond [3,10,11]. To address this period of high risk, the American College of Obstetricians and Gynecologists and the American Society of Addiction Medicine recommends that “*Access to…relapse prevention programs should be made available*” [6]. However, there are currently no evidence-based relapse prevention programs tailored to the unique postpartum circumstances [12]. Additionally, factors that contribute to the increased risk of return to use during postpartum are not well understood [9]. Ultimately, developing effective relapse prevention programs that can work adjunctive to MOUD and are tailored to postpartum-specific needs will allow parents, infants, and families enhanced opportunities to achieve positive long-term outcomes.

Ovarian hormones have been implicated as important factors for clinical treatment of substance use disorders [13-15]. For instance, in ovariectomized female rats offered intermittent access to fentanyl, those who received exogenous estradiol treatment exhibited increases in self-administration, enhanced sensitivity, and increased motivation versus placebo treatment [16]. Exogenous progesterone treatment is associated with reductions in combustible cigarette smoking during the postpartum period, as well as in nonpregnant females [17-19]. Hormonal influences on substance misuse behaviors may be amplified during the perinatal period, as hormones change by up to 300-fold during pregnancy, followed by a dramatic decline in the early postpartum and additional variation with lactation [20]. Interestingly, some of the natural hormonal patterns that occur during the perinatal period directly mirror the risk of substance misuse. For instance, both estrogen (which facilitates drug-taking behaviors) and risk of substance misuse are low during lactation, whereas without lactation, both estrogen and risk of substance misuse increase [20,21]. Further, specific infant caregiving activities can have substantial effects on hormones (eg, oxytocin increases during skin-to-skin contact), which may directly or indirectly impact postpartum substance relapse risk [22-25]. Despite the observed links between ovarian hormones and substance misuse as well as infant caregiving approaches and ovarian hormones, paired with the dramatic perinatal hormonal variations, the specific examination of postpartum hormonal patterns with OUD recovery-related outcomes has yet to be explored.

We conducted a prospective cohort study with those who did and did not have perinatal OUD to examine hormones or infant caregiving activities as modifiable risk factors that future interventions may target to enhance protection against return to opioid misuse. In order to specifically study postpartum relapse risk, we recruited participants with OUD who were stable in recovery during pregnancy, as well as comparable control group participants without OUD, and followed them across the high-risk postpartum period. As a first step in dissemination, this paper aims to (1) detail our protocols and methodologies and (2) report on recruitment, retention, compliance, and participant satisfaction. Ultimately, the goal of this paper is to enhance the understanding of perinatal OUD experience and inform the development of novel interventions and treatment options to support postpartum recovery and well-being.

## Methods

### Ethical Considerations

This study was approved by the University of Arizona’s Institutional Review Board (2009060851R001). Study staff met with all participants to obtain informed consent prior to their participation in the study. To maintain privacy and confidentiality, all data were stored with an alphanumeric identifier on secured platforms, only accessible by study staff. Participants were compensated up to US $725.

### Recruitment

In this prospective cohort study, we sought to enroll 50 pregnant people with OUD (OUD+) and 25 pregnant people without OUD (OUD–). We opted for a prospective cohort study design with frequent measurements to allow for the assessment of numerous modifiable risk factors (eg, estradiol, skin-to-skin contact). Specifically, with a primary sample of 75 independent participants who completed daily measurements across the first 12 weeks postpartum, this longitudinal study was designed to yield approximately 70,000 data points, providing adequate statistical power for within-person analyses and robust estimation of individual trajectories. It is noted that this sample size has limited power for between-group comparisons. However, the inclusion of OUD– will allow us to explore hormonal or infant caregiving approaches that are unique to OUD+. Overall, this approach will achieve the primary goal of preliminarily identifying meaningful relationships between hormones or infant caregiving with return to opioid misuse that can be further explored in future fully powered randomized control trials.

Participants met the following inclusion criteria: (1) 18‐40 years old, (2) uncomplicated single-gestation pregnancy at gestational week 30 or beyond, (3) a self-reported expectation of residing with the infant after birth, (4) English fluency, and (5) willing and able to comply with procedures. Additionally, OUD+ had to (6) report use of opioids during pregnancy, (7) report current OUD treatment with plans to continue treatment after childbirth, and (8) allow us access to their OUD treatment records. All participants were excluded for preterm birth (<36 gestational wk), planned long-distance moves, or extended travel through 6 months postpartum. Additional OUD– exclusion criteria included the use of opioids during pregnancy or a history of opioid use disorder. Finally, upon enrollment, we utilized frequency matching at the group level with a goal of ensuring the groups were comparable on proportion with no health insurance or public insurance versus private insurance, as well as on average age (ie,±5 y).

We recruited participants in the metro areas of Tucson and Phoenix, Arizona, from August 2021 to March 2024 via flyers or tabling at local health care provider clinics, community resources, community events, and social media. Individuals expressed their interest via a survey on REDCap [26], and this was followed by a telephone-based eligibility interview with staff. If eligibility criteria were met, potential participants were invited to a face-to-face baseline visit to commence participation.

### Data Collection Procedures

We implemented a data collection protocol similar to one we previously used with success in a pilot study focused on postpartum cigarette smoking relapse prevention [17]. Participants completed 15 visits either in-person (at our clinic or the participant’s home) or remotely via Health Insurance Portability and Accountability Act–compliant Zoom for Health, with all data entered on REDCap [26]. Visits were scheduled to begin between 7 AM and 11 AM given the known diurnal patterns in hormones and other variables of interest [27,28]. For those who attended remotely, study supplies were mailed in advance. All visits included the collection of biological samples, with all participants provided 1.8 mL of saliva via passive drool. Remote participants self-collected 12 drops of blood for dried blood spots using our previously developed protocol [29]. In-person participants provided 8 mL of venous blood, which was then processed to yield 12 drops of dried blood spots plus plasma samples. For remote participants, dried blood spots were mailed to staff weekly, and frozen saliva samples were picked up in batches by staff. All samples were subsequently stored at ≤−20 °C.

#### Baseline

At gestational week 36 or beyond, participants completed a baseline visit. After providing informed consent, participants completed an interview with staff to obtain their medical history, followed by completion of surveys and biological sample collection. Next, participants completed a semistructured audio-recorded interview with staff on lifetime trauma and resilience (Multimedia Appendix 1). At the end of the visit, participants were trained on how to complete daily surveys and at-home saliva samples, followed by receipt of compensation.

#### Daily Surveys

Beginning the day after baseline through postpartum week 12, participants completed daily surveys on their own or study-supplied smartphones. This was replicated the week preceding follow-up visits. Participants received a daily text message or email at 8 PM containing a link to surveys. Surveys remained open until 11:59 PM, with reminders every 30 minutes until completed. Surveys took approximately 10‐15 minutes to complete and documented use of and craving for substances, interactions, and connectedness with the infant and others, and other validated measures to capture return to use risk factors.

#### Fourth Trimester (Postpartum Weeks 1‐12)

Participants notified staff of childbirth via the daily surveys, then commenced weekly visits. At each visit, participants completed surveys and provided in-visit biological samples. Additionally, at-home saliva samples were collected at 8 PM the day before and 30 minutes after waking on the day of the visit. Participants were also interviewed to capture substance use, changes in health or medication, and time spent with the infant. At postpartum week 1, participants completed a semistructured audio-recorded interview with staff to capture birth and initial breastfeeding experiences (Multimedia Appendix 2). Approximately 1 year into study recruitment, we added a study satisfaction survey to postpartum weeks 1 and 12. Upon completion of each visit, the next visit was confirmed, and participants were compensated.

#### Follow-Up Visits (Postpartum Months 4 and 5)

Procedures were identical to the fourth trimester data collection. In addition, at postpartum month 5, participants completed a semistructured audio-recorded interview with staff to capture their breastfeeding experiences and a final satisfaction survey.

### Study Measures

Overall, participants completed a series of instruments on a daily, weekly, and monthly basis, as well as at follow-up (MultimediaAppendices 3  4).

#### Background Variables

During the enrollment interview and at the baseline visit, standardized questionnaires and interviews were used to capture sociodemographics (eg, age, race, ethnicity), medical history (eg, parity, diagnosis of hormone-influencing conditions), and history of substance use (eg, lifetime use of 16 different substances, drug of choice, OUD treatment experiences). The baseline interview incorporated Stressful Life Events [30], Adverse Childhood Experiences [31], Early Trauma Inventory [32,33], and Resilience Scale [34].

#### Maternal Factors

These variables included (1) known or suspected risk factors for postpartum substance misuse, (2) known or suspected association with hormones of interest, and (3) infant caregiving and parental experiences [35-51]. In brief, participants reported on lactation and breastfeeding (daily first 4 weeks, weekly thereafter), pain (daily first 4 weeks), and vaginal bleeding (daily after postpartum week 4), with additional assessments on a weekly or monthly basis.

#### Caregiving Factors

Variables included those that may contribute to risk of postpartum return to misuse or influence hormone levels. Daily, participants reported on their perceptions of parenting challenge, parenting reward, and connections with infant and with others using a 100-point visual analog scale ranging from “not at all” to “extremely.” Relative time spent with infant each day was reported using an investigator-created instrument with 100-point visual analog scale ranging from “a lot less than usual” to “a lot more than usual” to report on the amount of time spent: (1) in skin-to-skin contact with infant, (2) in other physical contact with infant, (3) caring for infant but not in physical contact, (4) with someone else while they cared for infant, and (5) time away from infant. Weekly staff interviewed participants to capture the average absolute time spent on each of these activities during the preceding week, ensuring that total time reported equated to 24 hours per day. Additional validated items were completed regularly throughout follow-up.

#### Hormones

We measured a total of 19 hormones at each time point. Cortisol and oxytocin assays were completed by the Laboratory for the Evolutionary Endocrinology of Primates at the University of Arizona. Saliva samples were stored at −80 °C, thawed to room temperature before analysis, and cleaned by centrifuging for 30 minutes at 3000 rpm with the supernatant retained. Saliva was aliquoted into microcentrifuge tubes at volumes of 500 µL for oxytocin and 150 µL for cortisol, then frozen at −80 °C until lyophilization. Samples were lyophilized at −80 °C and 0.3 mbar until completely dry, then resuspended in 250 µL and 150 µL of assay buffer (Arbor Assays, Ann Arbor, MI, USA), respectively. This resulted in 2x concentration for oxytocin and 1x concentration for cortisol. Samples were assayed according to protocols included with DetectX ELISA oxytocin (K048-H5; cross-reactivity with isotocin 94.3%, mesotocin 88.4%, other analytes <0.15%; sensitivity 17.0 pg/mL; limit of detection 22.9 pg/mL) and DetectX ELISA cortisol kits (K003-H5; cross-reactivity with dexamethasone 18.8%, prednisolone [1-dehydrocortisol] 7.8%, corticosterone and cortisone 1.2%, other analytes <0.1%; sensitivity 27.6 pg/mL; limit of detection 45.4 pg/mL). Plates were read at 450 nm on a BioTek Multi-Mode plate reader. The Laboratory for the Evolutionary Endocrinology of Primates conducted analytical validations of methods for adult female human saliva using tests of parallelism and accuracy for each analyte. Parallelism was done by serially diluting a sample pool, correcting the resulting concentrations at each dilution, and calculating the coefficient of variation [52]. The coefficient of variation of corrected concentrations from serially diluted saliva was 11.65% for oxytocin (n=5 dilutions) and 8.34% for cortisol (n=10 dilutions). For accuracy, sample pools were spiked with 10% synthetic oxytocin or cortisol in assay buffer, at different concentrations along the standard curve. Recovery for salivary oxytocin was 119.99%±0.05, and recovery for salivary cortisol was 102.18%±2.31. All samples were assayed in duplicate, and all plates included high and low pool controls to assess inter- and intra-assay variation. Any samples with concentrations that were outside of the standard curve range were diluted or concentrated and reassayed. Data were processed using the BioTek Gen5 software (version 3.08; Aligent Technologies) to calculate analyte concentrations.

Using dried blood spots, ZRT Laboratory (Beaverton, OR) measured cortisol, estrone, estradiol, estriol, testosterone, progesterone, DHEAS, cortisone, estrone-1-sulfate, pregnenolone sulfate, 17-hydroxyprogesterone, androstenedione, 7-keto DHEA, corticosterone, 11-deoxycortisol, ethinyl estradiol, anastrozole, and letrozole as previously described with some modifications [53]. Three 6-mm diameter punches were taken from each dried blood spot specimen and rehydrated. Steroids were extracted with organic solvent in the presence of internal standard, purified by solid-phase extraction, derivatized (estrogens only), and then analyzed by liquid chromatography-mass spectrometry. Liquid chromatography-mass spectrometry analysis was performed using ultra-fast liquid chromatography system (Shimadzu Nexera XR) coupled to a triple quadrupole mass spectrometer (AB Sciez 5500) equipped with an electrospray ionization source. Separations were conducted by reversed-phase chromatography, and the mass spectrometer was operated in multiple reaction monitoring mode. Data were processed using the Sciex OS software (version 1.7.2; AB Scienx) to determine analyte concentrations. All samples were run with dried blood spot controls, generated in-house by spiking stripped serum with various analyte concentrations, mixing washed red blood cells, and then spotting onto filter paper.

#### Factors Related to Substance Use

To assess postpartum risk for opioid misuse, we included 3 outcomes. First, daily self-reports of opioid cravings (separately for opioid-containing prescription medications, heroin, and MOUD medications) and coping with cravings. Both cravings and coping with craving were reported with a 100-point visual analog scale ranging from “not at all” to “severe” or “very well,” respectively. Second, daily self-reports of opioid use were captured via daily surveys and staff-administered TimeLine FollowBack [54] interviews at each visit. These approaches were also used to capture craving and use of 15 other substances. Finally, we reviewed toxicology results and treatment adherence via treatment program medical records.

### Statistical Analyses

Study sample descriptors (eg, sociodemographics, substance use) and outcomes were summarized with descriptive statistics. Group differences (ie, OUD+ vs OUD–) were compared using Welch *t* tests, *χ*^2^ tests, and Fisher exact tests. For the latter, although some cells contained relatively large counts (up to 45), exact *P* values were computed without the need for permutations. Group differences in compliance over time were also examined with binomial logistic regression models. All analyses were completed using open-source Python (3.10.11) libraries, Pandas 2.0.1 (NumFOCUS, Inc.), SciPy 1.13.0, and R Studio (2024.09.1, Build 394).

## Results

### Study Sample

**Figure 1.**
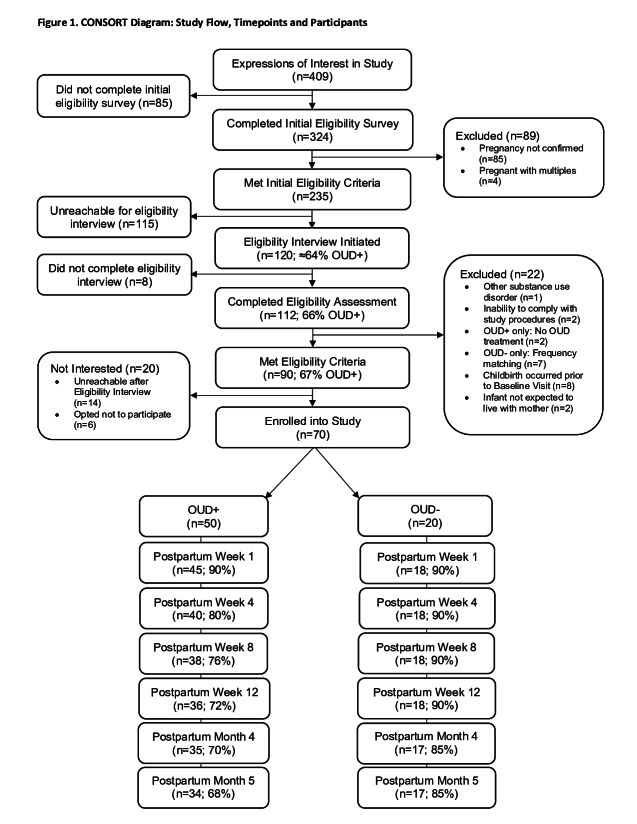
Consolidated Standards of Reporting Trials (CONSORT) diagram: study flow, time points, and participants. OUD: opioid use disorder.

A total of 70 participants enrolled in the study and completed the baseline assessment to form the final sample (Figure 1; note that these numbers exclude 1 participant who enrolled into the OUD– and later requested her data be removed). The final enrolled sample included 50 (71%) OUD+ and 20 (29%) OUD– (Table 1). Study groups were exchangeable on all sociodemographic variables except for gestational week prenatal care was initiated (OUD+: 9.8 [SD 5.2] vs OUD–: 7.1 [SD 3.8]; *P* value=.02). There was little evidence of selection bias from phone screening or enrollment to the postpartum data collection period (MultimediaAppendices 5  6). For example, the average age at enrollment was 28.6 (SD 5.2; n=112) compared to 29.4 (SD 5.1) among those who completed at least 1 postpartum visit (n=61). Additionally, 52% (n=58) of participants who completed the phone screening identified as Hispanic and 37% (n=41) reported education of some college compared to 51% (n=31) and 41% (n=25), respectively, at postpartum data collection. Among OUD+, at enrollment, 58% (n=50) reported being in recovery for less than a year compared to 56% (n=43) at postpartum data collection, with an age of first use of heroin reported as 17.3 (SD 5.7) years old (n=47) versus 17.5 (SD 5.9) years old (n=41), respectively.

**Table 1.** Description of final study sample (n=70).

	Total (n=70)	OUD+ (n=50)	OUD– (n=20)	Test statistic^a^ (*P* value)
Sociodemographic variables
Age (y), mean (SD)	29.3 (5.0)	30.0 (4.9)	27.8 (5.1)	1.63 (.11)
Race and ethnicity, n (%)	3.18 (.53)
Hispanic	33 (47)	21 (42)	12 (60)
NH^b^, White	28 (40)	21 (42)	7 (35)
NH, NA/AN^c^	4 (6)	3 (6)	1 (5)
NH, NH/PI^d^	0 (0)	0 (0)	0 (0)
NH, Asian	1 (1)	1 (2)	0 (0)
NH, B/AA^e^	4 (6)	4 (8)	0 (0)
Highest level of education completed, n (%)				9.11 (0.10)
≤8th grade	2 (3)	2 (4)	0 (0)
Some HS^f^	8 (11)	6 (12)	2 (10)
HS or equivalent	25 (36)	19 (38)	6 (30)
Some college or 2-year degree	30 (43)	22 (44)	8 (40)
College graduate or 4-year degree	3 (4)	0 (0)	3 (15)
Graduate or professional degree	2 (3)	1 (2)	1 (5)
Insurance status, n (%)	2.85 (.09)
Private	6 (9)	2 (4)	4 (20)
Public or none	64 (91)	48 (96)	16 (80)
Missing	0 (0)	0 (0)	0 (0)
Number of children living in home, mean (SD)	1.1 (1.4)	1.2 (1.6)	1.0 (0.9)	0.42 (.68)
Gestational week prenatal care initiated, mean (SD)	9.1 (5.0)	9.8 (5.2)	7.1 (3.8)	2.38 (.02)
Parity, n (%)	0.002 (.97)
Primiparous	19 (27)	13 (26)	6 (30)
Multiparous	51 (73)	37 (74)	14 (70)
Gestational week at baseline visit, mean (SD)	36.8 (0.8)	36.9 (0.9)	36.8 (0.6)	0.32 (.75)
Substance use history
Lifetime history of use of substances with abuse potential^g^, n (%)	—^h^
Opioid-containing prescription	58 (83)	48 (96)	10 (50)
Heroin	30 (43)	30 (60)	0 (0)
Narcotics	47 (67)	47 (94)	0 (0)
Fentanyl	6 (9)	6 (12)	0 (0)
Caffeine	68 (97)	48 (96)	20 (100)
Alcohol	60 (86)	43 (86)	17 (85)
Cigarettes or nicotine	56 (80)	43 (86)	13 (65)
Cannabis	56 (80)	45 (90)	11 (55)
Cocaine	44 (63)	41 (82)	3 (15)
Use in 3 months prior to pregnancy of substances with abuse potential^g^, n (%)	—
Opioid-containing prescription	18 (26)	18 (36)	0 (0)
Heroin	3 (4)	3 (6)	0 (0)
Narcotics	29 (41)	29 (58)	0 (0)
Fentanyl	4 (6)	4 (8)	0 (0)
Caffeine	66 (94)	46 (92)	20 (10)
Alcohol	27 (39)	12 (24)	15 (75)
Cigarettes or nicotine	44 (63)	34 (68)	10 (50)
Cannabis	38 (54)	31 (62)	7 (35)
Cocaine	9 (13)	7 (14)	2 (10)
Use in last 3 months of pregnancy of substances with abuse potential^g^, n (%)	—
Opioid-containing prescription	10 (1)	10 (20)	0 (0)
Heroin	0 (0)	0 (0)	0 (0)
Narcotics	41 (59)	41 (82)	0 (0)
Fentanyl	2 (3)	2 (4)	0 (0)
Caffeine	63 (90)	44 (88)	19 (95)
Alcohol	2 (3)	2 (4)	0 (0)
Cigarettes or nicotine	32 (46)	28 (56)	4 (20)
Cannabis	16 (23)	15 (30)	1 (5)
Cocaine	1 (1)	1 (2)	0 (0)
Drug of choice (top 5), n (%)	—
Other opioid-containing prescription	—	18 (36)	—
Heroin	—	11 (22)	—
Fentanyl	—	6 (12)	—
Cannabis	—	5 (10)	—
Caffeine	—	4 (8)	—
Age of first opioid-containing prescription use, mean (SD)	—	17.3 (5.7) (n=47)	—	—
Age of first heroin use, mean (SD)	—	22.4 (5.7) (n=30)	—	—
Lifetime history treatment type, n (%)	—
Inpatient	—	30 (60)	—
Outpatient	—	33 (66)	—
Intensive outpatient	—	27 (54)	—
Current treatment type, n (%)	—
Inpatient	—	2 (4)	—
Outpatient	—	30 (60)	—
Intensive outpatient	—	5 (10)	—
Current treatment components, n (%)	—
Medications	—	34 (68)	—
Counseling/support groups	—	24 (48)	—
Other	—	3 (6)	—
Recovery length, n (%)	—
>1 year	—	21 (42)	—
Before this pregnancy but <1 year	—	9 (18)	—
First trimester of this pregnancy	—	6 (12)	—
Second trimester of this pregnancy	—	10 (20)	—
Third trimester of this pregnancy	—	4 (8)	—

a Test statistic value listed is *χ*2 or *t* value.

b NH: Non-Hispanic.

c NA/AN: Native American or Alaskan Native.

d NH/PI: Native Hawaiian or Pacific Islander.

e B/AA: Black or African American.

f HS: high school.

g Substances listed include opioids and the 5 most commonly endorsed substances used per lifetime history.

h —: not applicable.

### Protocol Compliance

**Figure 2.**
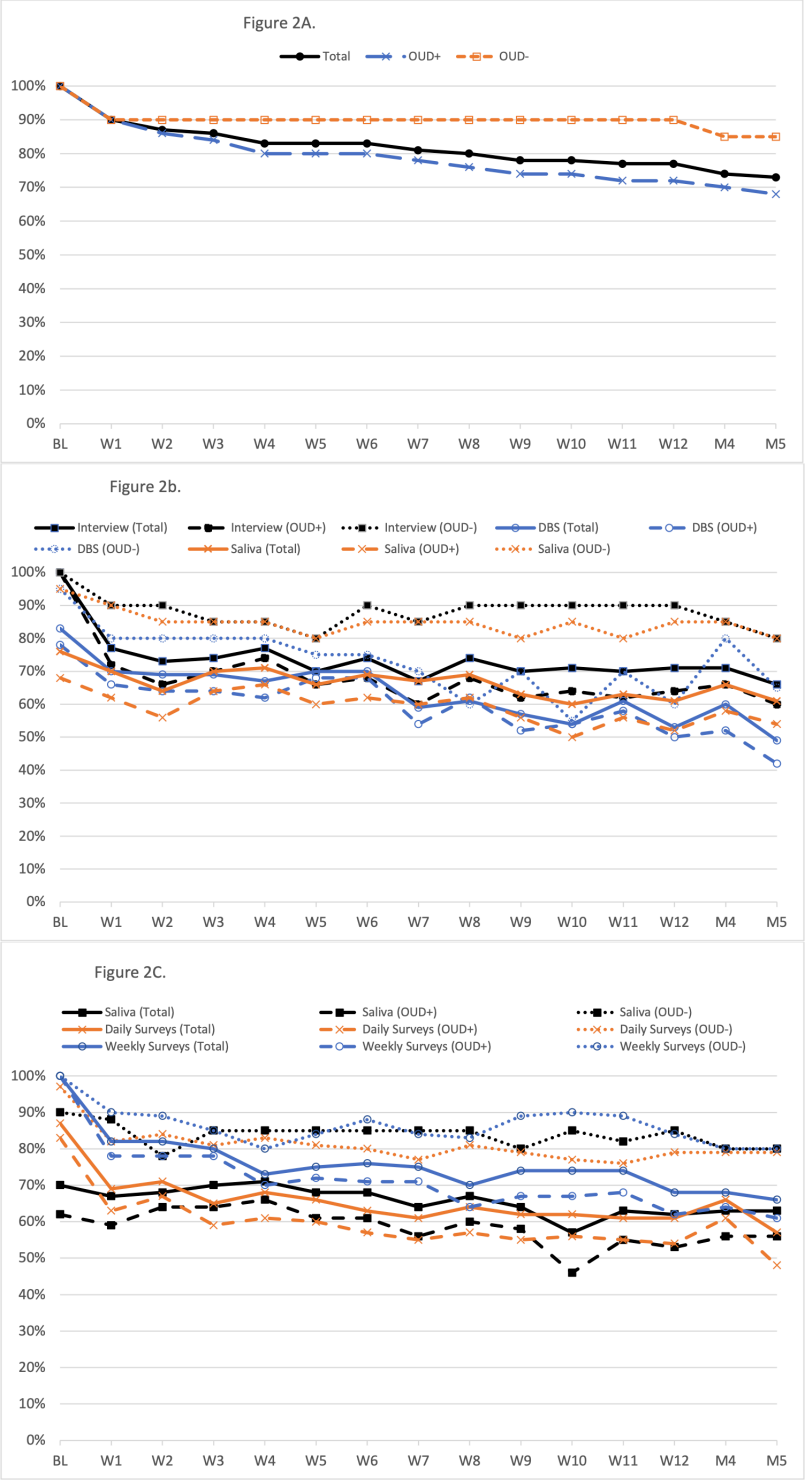
Completion rates by study group, time point, and procedure: (a) visit completion by study group, (b) compliance with in-visit procedure over time by modality and group, and (c) compliance with out-of-visit procedure over time by modality and group. OUD: opioid use disorder.

Nearly all participants (n=65, 93%) reported childbirth to staff (OUD+: 92%, OUD–: 95%), and 62 (87%) completed at least 1 study visit post-childbirth (OUD+: 86%, OUD–: 90%). Visit completion rates post-baseline ranged from 90% at postpartum week 1% to 73% at postpartum month 5 (Figure 2; Multimedia Appendix 7), with lower rates in the OUD+ group and at later time points. Compliance with procedures followed similar patterns (lower in OUD+ and at later time points), with the highest overall compliance, on average, with the weekly surveys (81%), followed by interviews (74%), saliva samples (66%), daily surveys (66%), and dried blood spots (63%). While there were no differences in the decline in compliance over time by group by procedure (interviews [*β*=.04, *P*=.40], dried blood spots [*β*=.04, *P*=.22], saliva samples [in visit: *β*=.01, *P*=.89; outside of visit: *β*=−.01, *P*=.82], nor surveys [daily: *β*=−.01, *P*=.80; weekly: *β*=−.04, *P*=.10]), OUD+ visit attendance declined more rapidly over time compared to OUD– (*β*=−.06, *P*=.03).

### Study Satisfaction

Among the subgroup of participants who completed the study satisfaction surveys, results indicate that the study was well tolerated over time with no significant differences by study groups (Table 2).

**Table 2.** Study satisfaction by study group and time point^a^.

	Total	OUD+	OUD–	*P* value^b^
Sample size by time point^c^				
W1^d^	20	17	3	—^e^
W12^f^	31	25	6	—
M5^g^	45	32	13	—
Based on your experience, how willing would you be to participate in a study like this again? Mean (SD)				
W1	2.6 (0.8)	2.6 (0.9)	2.7 (0.6)	.60
W12	2.9 (0.4)	2.8 (0.5)	3.0 (0.0)	>.99
M5	2.7 (0.8)	2.7 (0.8)	2.7 (0.6)	.56
Overall, how much of a burden has it been to participate in this study? Mean (SD)				
W1	0.6 (0.8)	0.6 (0.9)	0.3 (0.6)	>.99
W12	0.5 (0.8)	0.6 (0.9)	0.3 (0.5)	>.99
M5	0.4 (0.7)	0.4 (0.8)	0.4 (0.6)	>.99
How much of a burden was it to participate in this study before your baby was born? Mean (SD)				
W12	0.2 (0.6)	0.2 (0.6)	0.0 (0.0)	>.99
How much of a burden was it to participate in this study after your baby was born? Mean (SD)				
W12	0.6 (0.8)	0.6 (0.9)	0.5 (0.5)	.51
Overall, how much has participating in this study interfered with your usual activities? Mean (SD)				
W1	0.7 (0.8)	0.7 (0.8)	0.3 (0.6)	>.99
W12	0.6 (0.8)	0.5 (0.8)	1.0 (1.1)	.38
How much did participating before your baby was born interfere with your usual activities? Mean (SD)				
W12	0.2 (0.6)	0.3 (0.7)	0.2 (0.4)	>.99
How much did participating after your baby was born interfere with your usual activities? Mean (SD)				
W12	0.7 (0.8^h^)	0.7 (0.9^h^)	0.7 (0.5)	.53
How much difficulty did you have completing the study questionnaires? Mean (SD)				
W1	0.3 (0.7)	0.4 (0.8)	0.0 (0.0)	>.99
How much difficulty did you have using a cell phone to complete the study questionnaires? Mean (SD)				
W12	0.1 (0.4)	0.1 (0.4)	0.0 (0.0)	>.99
How much have you been annoyed with the number of alerts you have received in this study before your baby was born? Mean (SD)				
W12	0.2 (0.5^h^)	0.3 (0.5)	0.0 (0.0^h^)	.63
How much have you been annoyed with the number of alerts you have received in this study after your baby was born? Mean (SD)				
W12	0.3 (0.7)	0.4 (0.8)	0.2 (0.4)	>.99
How difficult was it to notify the study staff of your child’s birth? Mean (SD)				
W1	0.1 (0.7)	0.2 (0.7)	0.0 (0.0)	>.99
Have you had any difficulties working with study staff? Mean (SD)				
W1	0.0 (0.0)	0.0 (0.0)	0.0 (0.0)	—
W12	0.0 (0.0)	0.0 (0.0)	0.0 (0.0)	—
M5	0.0 (0.2)	0.1 (0.2)	0.0 (0.0)	>.99

a Values are as follows: 0: not at all, 1: slightly, 2: moderately, 3: extremely.

b Fisher exact test.

c Adding percentages would cause confusion (eg, it could imply percent of full sample, percent at time point, or percent of group). Hence, the sample size is listed without percentages in the table.

d W1: postpartum week 1.

e —: not applicable.

f W12: postpartum week 2.

g M5: postpartum month 5.

h One participant did not respond to this item.

## Discussion

### Principal Results

We completed a prospective cohort study with individuals who did and did not have perinatal OUD, following them with robust data collection procedures. This included daily surveys and weekly face-to-face visits with collection of biological samples during the first 12 weeks postpartum with additional follow-up through postpartum month 5. We enrolled 70 participants during late pregnancy, and 51 (73%) completed the follow-up through postpartum month 5. Notably, more participants in the OUD– group completed follow-up compared to the OUD+ group (85% vs 68%, respectively). Additionally, despite the challenges faced in caring for a newborn paired with our robust data collection protocol, compliance with specific data collection modalities was moderately high in both groups (63%‐81%). Indeed, the daily survey compliance of approximately 80% during the follow-up period among OUD– is directly in line with the, on average, 80% compliance rates (range: 63%‐96%) observed in 27 studies using similar procedures during the perinatal period [55]. The OUD+ group had lower compliance overall, throughout the follow-up period for the data collection procedures, as well as a faster drop in compliance over time with regard to visit attendance. This is to be expected given the additional demands on the OUD+ participants (eg, caring for a high-needs infant, daily MOUD clinic visits). Moreover, during the first year of data collection, all participants faced additional challenges with the ongoing COVID-19 pandemic. Despite this, among a subset of participants, high levels of satisfaction were reported in both groups consistently across time. While this data collection protocol was burdensome with daily surveys plus weekly meetings and hormonal measurements and, thus, likely includes some selection bias due to the nature of individuals who are able to participate in this research (eg, those with more support at home, those more stable in recovery), it does provide an opportunity to inform future research on relapse prevention development.

The primary goal of this prospective cohort study is to examine the potential utility of hormones or infant caregiving activities to support postpartum OUD recovery (publications forthcoming). While there is growing evidence that hormones (eg, progesterone, estrogen, oxytocin) influence drug-taking behaviors, nearly all research has examined the effect of a single hormone in isolation at 1 or 2 time points [13-15]. Emerging research demonstrates that relative levels and temporal patterns of hormones may be more important than a single hormone value at an isolated time point. For example, changes in the ratio of progesterone to estradiol across two time points are more predictive of smoking behavior than either hormone alone at a single time point [56]. Given that hormones (1) are continuously in flux, especially during the perinatal period, and variations occur as a result of many factors, including specific infant caregiving activities, and (2) have a significant effect on several aspects of neurobiology, including drug reward and stress responses, assessing the effects of 1 or 2 hormones at 1 or 2 time points is insufficient.

With the daily surveys and weekly hormonal measurements, we will be able to examine the role of hormones and infant caregiving practices on OUD recovery-related outcomes with novel analytical approaches to model complex variable interactions both concurrently and across time. Forthcoming analyses addressing this goal will follow recommendations to handle missing data in longitudinal datasets, including the use of multilevel modeling frameworks that use all available data across repeated measures and maximum likelihood and imputation methods for relatively small sample sizes (eg, full information maximum likelihood, joint multiple imputation) [57-59]. Further, we will employ sensitivity analyses to assess potential selection bias and mitigate error [60-63] to further approximate observations to the target population.

While this study may not produce generalizable results, it can provide direction for future research. For example, if our observations suggest that higher levels of progesterone are protective against relapse risk, exogenous progesterone may be used to favorably modify progesterone during the postpartum period to reduce relapse risk, as was recently done to reduce postpartum cigarette smoking relapse risk [17]. Another scenario may include our observations suggesting that more close contact with infants is protective against postpartum opioid craving, in line with our prior work demonstrating that babywearing is linked to reduced urges to use substances [56]. Consequently, a follow-up study may examine the results of maternal babywearing on postpartum recovery outcomes similar to recent studies with nurses and mothers caring for infants with neonatal abstinence syndrome [64,65]. A third example may be heightened estradiol or testosterone during postpartum may be linked to more cue-induced cravings. While the exogenous delivery of these hormones is not compatible with pregnancy or breastfeeding [66,67], future research may instead use innovative approaches to monitor hormone pattern(s) or level(s) remotely [68] to proactively identify periods of heightened risk leading to the deployment of additional supports. This approach could also be applied to remotely monitor infant caregiving via smart baby carriers [68]. In sum, the data resulting from the study protocol described here, while limited, will yield informative preliminary results that can be further examined via a priori hypothesis testing approaches (eg, randomized control trials) with fully powered and diverse study samples to yield generalizable results and inform the development of additional postpartum OUD recovery support.

Beyond our primary goals, these data provide ample opportunities to explore novel topics in perinatal substance misuse and beyond. For example, we conducted comprehensive interviews of lifetime trauma exposure and resilience, as well as birth and breastfeeding experiences. This allows for mixed-method approaches to expand the understanding of these experiences prospectively during the perinatal period. Additionally, daily our participants reported on their mood, affect, and stress, as well as their craving for and use of other substances (eg, alcohol, cannabis, nicotine, or tobacco). Thus, we can explore the relationships between these variables, as well as in relation to baseline characteristics (eg, ethnicity, parity) and concurrent behaviors and activities (eg, sleep, involvement of others). Indeed, we are currently exploring the role of loneliness, isolation, and social support in OUD-related outcomes within this dataset (R21DA058364; Allen and Linde-Krieger). Overall, these data offer substantial opportunities to uncover novel relationships, increase our understanding of risk or protective factors of maladaptive behaviors during the sensitive perinatal period, and ultimately inform new approaches to enhance the health and well-being of mothers, infants, and families.

### Limitations

Like every study, there are limitations. First, generalizability is limited given that these data were collected in a single US state, and the robust protocol may have been too burdensome for some individuals. Similarly, our eligibility criteria restricted our OUD+ group to those who were in treatment for recovery and expected to live with their infant. It is also possible that those who had lower cravings or better coping were less likely to discontinue their study participation. We also have a fair amount of missing data, as is common in prospective studies during the postpartum period with daily measurements [55]. These data may not be missing at random (eg, those with a high-needs newborn or those at higher risk for return to opioid misuse may be less likely to complete data collection; retention may have changed across the study due to the COVID-19 pandemic or protocol adjustments). It is possible that this will introduce bias and error into our observations. Finally, this study is underpowered to test hypotheses using traditional analytical approaches for causal modeling. However, our goal here is to generate new preliminary observations that will lead to fully powered trials that can adequately assess these relationships with diverse study samples and that utilize novel approaches to prevent opioid misuse. Despite these limitations, there are also substantial strengths, including an ability to temporally evaluate relationships; assessment of substance use with multiple sources (eg, prospective surveys, retrospective interviews); and numerous validated measures with rigorous, gold-standard approaches.

### Conclusions

Overall, this prospective cohort study of those with and without OUD during the perinatal period resulted in moderately high retention and compliance, as well as high participant-reported study satisfaction, among a subgroup of participants. Consequently, these multidimensional data have high potential to inform future research on the challenges and opportunities of the perinatal period to support OUD recovery during the traditionally high-risk postpartum period.

## Supplementary material

10.2196/77899 Multimedia Appendix 1 Lifetime trauma and resilience interview.

10.2196/77899 Multimedia Appendix 2 Investigator-created birth and breastfeeding interview.

10.2196/77899 Multimedia Appendix 3 Protocol completion by time point, modality, and group.

10.2196/77899 Multimedia Appendix 4 Investigator-created and modified versions of validated instruments.

10.2196/77899 Multimedia Appendix 5 Overview of study measures.

10.2196/77899 Multimedia Appendix 6 Description of study sample—completed phone screening interview (n=112).

10.2196/77899 Multimedia Appendix 7 Description of study sample—completed at least 1 postpartum visit (n=61).

## Abbreviations

MOUD: medications for opioid use disorder
OUD: opioid use disorder

## References

[1] Haight SC, Ko JY, Tong VT, Bohm MK, Callaghan WM (2018). Opioid use disorder documented at delivery hospitalization—United States, 1999-2014. MMWR Morb Mortal Wkly Rep.

[2] Hirai AH, Ko JY, Owens PL, Stocks C, Patrick SW (2021). Neonatal abstinence syndrome and maternal opioid-related diagnoses in the US, 2010-2017. JAMA.

[3] Auty SG, Frakt AB, Shafer PR, Stein MD, Gordon SH (2025). Severe maternal morbidity among pregnant people with opioid use disorder enrolled in Medicaid. JAMA Netw Open.

[4] Terplan M, Martin CE, Premkumar A, Krans EE, Wakeman SE, Rich JD (2021). Treat Opioid Use Disord Gen Med Settings.

[5] Winstanley EL, Stover AN (2019). The impact of the opioid epidemic on children and adolescents. Clin Ther.

[6] Opioid Use and Opioid Use Disorder in Pregnancy.

[7] Schiff DM, Nielsen T, Terplan M (2018). Fatal and nonfatal overdose among pregnant and postpartum women in Massachusetts. Obstet Gynecol.

[8] Correlates of treatment retention and opioid misuse among postpartum women in methadone treatment.

[9] Frankeberger J, Jarlenski M, Krans EE, Coulter RWS, Mair C (2023). Opioid use disorder and overdose in the first year postpartum: a rapid scoping review and implications for future research. Matern Child Health J.

[10] Klaman SL, Isaacs K, Leopold A (2017). Treating women who are pregnant and parenting for opioid use disorder and the concurrent care of their infants and children: literature review to support national guidance. J Addict Med.

[11] Piske M, Homayra F, Min JE (2021). Opioid use disorder and perinatal outcomes. Pediatrics.

[12] Martinez A, Allen A (2020). A review of nonpharmacological adjunctive treatment for postpartum women with opioid use disorder. Addict Behav.

[13] Anker JJ, Carroll ME (2011). Females are more vulnerable to drug abuse than males: evidence from preclinical studies and the role of ovarian hormones. Curr Top Behav Neurosci.

[14] McHugh RK, Votaw VR, Sugarman DE, Greenfield SF (2018). Sex and gender differences in substance use disorders. Clin Psychol Rev.

[15] McKee SA, McRae-Clark AL (2022). Consideration of sex and gender differences in addiction medication response. Biol Sex Differ.

[16] Towers EB, Setaro B, Lynch WJ (2023). Estradiol enhances the development of addiction-like features in a female rat model of opioid use disorder. Neuroendocrinology.

[17] Allen SS, Allen AM, Lunos S, Tosun N (2016). Progesterone and postpartum smoking relapse: a pilot double-blind placebo-controlled randomized trial. Nicotine Tob Res.

[18] Tosun NL, Fieberg AM, Eberly LE (2019). Exogenous progesterone for smoking cessation in men and women: a pilot double-blind, placebo-controlled randomized clinical trial. Addiction.

[19] Forray A, Gilstad-Hayden K, Suppies C, Bogen D, Sofuoglu M, Yonkers KA (2017). Progesterone for smoking relapse prevention following delivery: a pilot, randomized, double-blind study. Psychoneuroendocrinology.

[20] Mishell DR (1999). Reproductive Endocrinology.

[21] Forray A, Merry B, Lin H, Ruger JP, Yonkers KA (2015). Perinatal substance use: a prospective evaluation of abstinence and relapse. Drug Alcohol Depend.

[22] Zeki S (2007). The neurobiology of love. FEBS Lett.

[23] Carter CS (1998). Neuroendocrine perspectives on social attachment and love. Psychoneuroendocrinology.

[24] Kenkel WM, Paredes J, Yee JR, Pournajafi‐Nazarloo H, Bales KL, Carter CS (2012). Neuroendocrine and behavioural responses to exposure to an infant in male prairie voles. J Neuroendocrinology.

[25] Kovatsi L, Nikolaou K (2019). Opioids and the hormone oxytocin. Vitam Horm.

[26] Harris PA, Taylor R, Thielke R, Payne J, Gonzalez N, Conde JG (2009). Research Electronic Data Capture (REDCap)—a metadata-driven methodology and workflow process for providing translational research informatics support. J Biomed Inform.

[27] Elverson CA, Wilson ME (2005). Cortisol: circadian rhythm and response to a stressor. Newborn Infant Nurs Rev.

[28] Forsling ML (2000). Diurnal rhythms in neurohypophysial function. Exp Physiol.

[29] Allen AM, Lundeen K, Murphy SE, Spector L, Harlow BL (2018). Web-delivered multimedia training materials for the self-collection of dried blood spots: a formative project. JMIR Form Res.

[30] Newton RW, Webster PA, Binu PS, Maskrey N, Phillips AB (1979). Psychosocial stress in pregnancy and its relation to the onset of premature labour. Br Med J.

[31] Felitti VJ, Anda RF, Nordenberg D (1998). Relationship of childhood abuse and household dysfunction to many of the leading causes of death in adults. Am J Prev Med.

[32] Bremner JD, Vermetten E, Mazure CM (2000). Development and preliminary psychometric properties of an instrument for the measurement of childhood trauma: the Early Trauma Inventory. Depress Anxiety.

[33] Bremner JD, Bolus R, Mayer EA (2007). Psychometric properties of the Early Trauma Inventory-Self Report. J Nerv Ment Dis.

[34] Wagnild GM, Young HM (1993). Development and psychometric evaluation of the Resilience Scale. J Nurs Meas.

[35] Cleeland CS, Ryan KM (1994). Pain assessment: global use of the Brief Pain Inventory. Ann Acad Med Singap.

[36] Cox JL, Holden JM, Sagovsky R (1987). Detection of postnatal depression. Development of the 10-item Edinburgh Postnatal Depression Scale. Br J Psychiatry.

[37] Park ER, Psaros C, Traeger L (2015). Development of a postpartum stressor measure. Matern Child Health J.

[38] Osman A, Wong JL, Bagge CL, Freedenthal S, Gutierrez PM, Lozano G (2012). The Depression Anxiety Stress Scales-21 (DASS-21): further examination of dimensions, scale reliability, and correlates. J Clin Psychol.

[39] Brockington IF, Fraser C, Wilson D (2006). The Postpartum Bonding Questionnaire: a validation. Arch Womens Ment Health.

[40] Crncec R, Barnett B, Matthey S (2008). Development of an instrument to assess perceived self-efficacy in the parents of infants. Res Nurs Health.

[41] Johns MW (1991). A new method for measuring daytime sleepiness: the Epworth sleepiness scale. Sleep.

[42] Buysse DJ, Reynolds CF, Monk TH, Berman SR, Kupfer DJ (1989). The Pittsburgh sleep quality index: a new instrument for psychiatric practice and research. Psychiatry Res.

[43] Russell DW (1996). UCLA Loneliness Scale (Version 3): reliability, validity, and factor structure. J Pers Assess.

[44] Sherbourne CD, Stewart AL (1991). The MOS social support survey. Social Science & Medicine.

[45] Barkin JL, Wisner KL, Bromberger JT, Beach SR, Terry MA, Wisniewski SR (2010). Development of the Barkin index of maternal functioning. J Womens Health (Larchmt).

[46] Ohan JL, Leung DW, Johnston C (2000). The Parenting Sense of Competence scale: evidence of a stable factor structure and validity. Can J Behav Sci.

[47] Luyten P, Mayes LC, Nijssens L, Fonagy P (2017). The parental reflective functioning questionnaire: development and preliminary validation. PLoS One.

[48] Fonagy P, Luyten P, Moulton-Perkins A (2016). Development and validation of a self-report measure of mentalizing: the reflective functioning questionnaire. PLOS ONE.

[49] Gartstein MA, Rothbart MK (2003). Studying infant temperament via the Revised Infant Behavior Questionnaire. Infant Behav Dev.

[50] Sadeh A (2004). A brief screening questionnaire for infant sleep problems: validation and findings for an Internet sample. Pediatrics.

[51] Wood RG, Moore Q, Clarkwest A, Killewald A, Monahan S (2019). The building strong families project: the long-term effects of building strong families: a relationship skills education program for unmarried parents.

[52] Plikaytis BD, Holder PF, Pais LB, Maslanka SE, Gheesling LL, Carlone GM (1994). Determination of parallelism and nonparallelism in bioassay dilution curves. J Clin Microbiol.

[53] Morimoto LM, Zava D, McGlynn KA (2018). Neonatal hormone concentrations and risk of testicular germ cell tumors (TGCT). Cancer Epidemiol Biomarkers Prev.

[54] Sobell LC, Cunningham JA, Sobell MB (1996). Recovery from alcohol problems with and without treatment: prevalence in two population surveys. Am J Public Health.

[55] Zhang X, Cui W, Yao J, Zhang Y, Wang Y (2025). Feasibility and utility of ecological momentary assessment to measure mental health issues in perinatal women: scoping review. Psychiatry Res.

[56] Schiller CE, Saladin ME, Gray KM, Hartwell KJ, Carpenter MJ (2012). Association between ovarian hormones and smoking behavior in women. Exp Clin Psychopharmacol.

[57] Grund S, Lüdtke O, Robitzsch A (2019). The Handbook of Multilevel Theory, Measurement, and Analysis.

[58] McNeish D (2017). Missing data methods for arbitrary missingness with small samples. J Appl Stat.

[59] Lee T, Shi D (2021). A comparison of full information maximum likelihood and multiple imputation in structural equation modeling with missing data. Psychol Methods.

[60] Infante-Rivard C, Cusson A (2018). Reflection on modern methods: selection bias—a review of recent developments. Int J Epidemiol.

[61] Robins JM, Rotnitzky A, Scharfstein DO, Halloran ME, Berry D (2000). Statistical Models in Epidemiology, the Environment, and Clinical Trials.

[62] Yaseliani M, Noor-E-Alam M, Hasan MM (2024). Mitigating sociodemographic bias in opioid use disorder prediction: fairness-aware machine learning framework. JMIR AI.

[63] Chung PC, Lin IF (2023). Sensitivity analysis of selection bias: a graphical display by bias-correction index. PeerJ.

[64] Rankin L, Grisham LM, Mendoza N, Allen A (2024). Babywearing reduces urges to use substances in the postpartum period among mothers with OUDs. Subst Use Misuse.

[65] Williams LR, Gebler-Wolfe M, Grisham LM, Bader MY (2020). “Babywearing” in the NICU. Adv Neonatal Care.

[66] Estradiol (Briggs Drugs in Pregnancy and Lactation) - UpToDate® LexidrugTM.

[67] Testosterone (Briggs Drugs in Pregnancy and Lactation) - UpToDate® LexidrugTM.

[68] Ye C, Wang M, Min J (2024). A wearable aptamer nanobiosensor for non-invasive female hormone monitoring. Nat Nanotechnol.

