# Supplementary material for: Compliance and Satisfaction With a Protocol for Identifying Novel Targets to Support Postpartum Opioid Use Disorder Recovery: Prospective Cohort Study
Source: JMIR Form Res. 2025 Nov 20;9:e77899. doi: 10.2196/77899 (PMC12633836; doi:10.2196/77899)
Supplement: Multimedia Appendix 4 [file formative-v9-e77899-s004.docx]

**Supplementary Document 4. Overview of Study Measures**

|  | **Timepoint of Collection** | **Mode of Collection** | **Location of Collection** | **Reference and/or  Supplement Document** |
| --- | --- | --- | --- | --- |
| **Background Variables** |  |  |  |  |
| Sociodemographics | Enrollment and Baseline | Self-Administered Survey and Interview with Staff | At home and during visit | Supplementary Document 3 |
| Medical History | Enrollment and Baseline | Self-Administered Survey and Interview with Staff | At home and during visit | Supplementary Document 3 |
| Substance Use History | Enrollment and Baseline | Self-Administered Survey and Interview with Staff | At home and during visit | Supplementary Document 3 |
| Stressful Life Events | Baseline | Audio-Recorded Interview with Staff | During visit | Newton et al [31],  Supplementary Document 1 |
| Adverse Childhood Experiences | Baseline | Audio-Recorded Interview with Staff | During visit | Felitti et al [32],  Supplementary Document 1 |
| Modified Early Trauma Inventory | Baseline | Audio-Recorded Interview with Staff | During visit | Bremner et al [33, 34]  Supplementary Document 1 |
| Resilience Scale | Baseline | Audio-Recorded Interview with Staff | During visit | Wagnild et al [35]  Supplementary Document 1 |
| Birth Interview | PW1 | Audio-Recorded Interview with Staff | During visit | Supplementary Document 2 |
|  |  |  |  |  |
| **Maternal Variables** |  |  |  |  |
| Breastfeeding/Lactation | Daily PW 1-4, Weekly PW 5-12, PM 4, PM 5 | Self-Administered Survey and Audio-Recorded Interview with Staff | At home and during visit | Supplementary Document 2 and 3 (Daily Assessment) |
| Vaginal Bleeding | Daily PW 5-12, PM 4, PM 5 | Self-Administered Survey | At home | Supplementary Document 3 (Daily Assessment) |
| Modified Brief Pain Inventory, Pregnancy and Postpartum Pain Inventory | Baseline, Weekly PW 1-4 | Self-Administered Survey | At home | Cleeland et al [36],  Supplementary Document 3 |
| Edinburgh Postnatal Depression Scale | Baseline, Weekly PW 1-12, PM 4, PM 5 | Self-Administered Survey | At home | Cox et al [37] |
| Modified Postpartum Stressor Scale | Baseline, Weekly PW 1-12, PM 4, PM 5 | Self-Administered Survey | At home | Park et al [38]  Supplementary Document 3 |
| Depression Anxiety Stress Scale | Baseline, Weekly PW 1-12, PM 4, PM 5 | Self-Administered Survey | At home | Osman et al [39] |
| Postpartum Bonding Questionnaire | Baseline, Weekly PW 1-12, PM 4, PM 5 | Self-Administered Survey | At home | Brockington et al [40] |
| Karitane Parenting Confidence Scale | Baseline, Weekly PW 1-12, PM 4, PM 5 | Self-Administered Survey | At home | Črnčec et al [41] |
| Modified Epworth Sleepiness Scale | Baseline, Weekly PW 1-12, PM 4, PM 5 | Self-Administered Survey | At home | Johns et al [42] Supplementary Document 3 |
| Modified Pittsburgh Sleep Quality Index | Baseline, Monthly PW 1-12, PM 4, PM 5 | Self-Administered Survey | At home | Buysse et al [43]  Supplementary Document 3 |
| UCLA Loneliness Scale-Revised | Baseline, Monthly PW 1-12, PM 4, PM 5 | Self-Administered Survey | At home | Russell et al^46^ |
| MOS Social Support Scale | Baseline, Monthly PW 1-12, PM 4, PM 5 | Self-Administered Survey | At home | Sherbourne et al [45] |
| Barkin Index of Maternal Functioning | Baseline, Monthly PW 1-12, PM 4, PM 5 | Self-Administered Survey | At home | Barkin et al [46] |
| Reflective Functioning Questionnaire | Baseline | Self-Administered Survey | At home | Fonagy et al [49] |
| Parental Reflective Functioning Questionnaire | PW 12, PM 4, PM 5 | Self-Administered Survey | At home | Luyten et al [48] |
|  |  |  |  |  |
| **Caregiving Variables** |  |  |  |  |
| Time Spent with Infant | Daily and Weekly PW 1-12, PM 4, PM 5 | Self-Administered Survey and Interview with Staff | At home and during visit | Supplementary Document 3 (Daily Assessment) |
| Subjective Response to Parenting | Daily PW 1-12, PM 4, PM 5 | Self-Administered Survey | At home | Supplementary Document 3 (Daily Assessment) |
| Infant Behavior Questionnaire | Weekly PW 1-12, PM 4, PM 5 | Self-Administered Survey | At home | Gradstein et al [50] |
| Modified Brief Infant Sleep Questionnaire | Weekly PW 1-12, PM 4, PM 5 | Self-Administered Survey | At home | Sadeh et al [51]  Supplementary Document 3 |
| Parenting Sense of Competency Scale | Monthly PW 1-12, PM 4, PM 5 | Self-Administered Survey | At home | Ohan et al [47] |
| Modified Father and Other Involvement Survey | Monthly PW 1-12, PM 4, PM 5 | Self-Administered Survey | At home | Wood et al [52]  Supplementary Document 3 |
|  |  |  |  |  |
| **Hormones** |  |  |  |  |
| Oxytocin | Baseline, Weekly PW 1-12, PM 4, PM 5 | Saliva Sample | During visit | - |
| Cortisol | Baseline, Weekly PW 1-12, PM 4, PM 5 | Saliva Sample | At home (8pm and 30 minutes after waking) | - |
| Cortisol, Estrone, Estradiol, Estriol, Testosterone, Progesterone, DHEAS, Cortison, Estrone-1-sulfate, Pregnenlone Sulfate, 17-hydroxyprogesterone, Androstenedione, 7-Keto DHEA, Corticosterone, 11-Dexycortisol, Ethinyl Estradiol, Anastrozole, Letrozole | Baseline, Weekly PW 1-12, PM 4, PM 5 | Dried Blood Spots | During visit | - |
|  |  |  |  |  |
| **Outcome Variables** |  |  |  |  |
| Craving | Baseline, Daily PW 1-12, PM 4, PM 5 | Self-Administered Survey | At home | Supplementary Document 3 (Daily Assessment) |
| Urge Coping | Baseline, Daily PW 1-12, PM 4, PM 5 | Self-Administered Survey | At home | Supplementary Document 3 (Daily Assessment) |
| Use (Prospective) | Baseline, Daily PW 1-12, PM 4, PM 5 | Self-Administered Survey | At home | Supplementary Document 3 (Daily Assessment) |
| Use (Retrospective via TimeLine FollowBack) | Baseline, Weekly PW 1-12, PM 4, PM 5 | Interview with Staff | During visit | Sobell et al [55]  Supplementary Document 3 (Daily Assessment) |
| Toxicology Results | Childbirth to One Year Postpartum | Medical Record Chart Review | n/a | - |
| Treatment Program Adherence | Childbirth to One Year Postpartum | Medical Record Chart Review | n/a | - |
