# Supplementary material for: Compliance and Satisfaction With a Protocol for Identifying Novel Targets to Support Postpartum Opioid Use Disorder Recovery: Prospective Cohort Study
Source: JMIR Form Res. 2025 Nov 20;9:e77899. doi: 10.2196/77899 (PMC12633836; doi:10.2196/77899)
Supplement: Multimedia Appendix 5 [file formative-v9-e77899-s005.docx]

**Supplementary Document 5. Description of Study Sample – Completed Phone Screening Interview (n=112)**

|  | **Total**  **(n=112)** | **OUD+ (n=74)** | **OUD- (n=38)** | **Test Statistic**^1^ **(p-value)** |
| --- | --- | --- | --- | --- |
| Age (mean ± standard deviation) | 28.6±5.2 | 28.8±5.2 | 28.1±5.1 | 0.72 (0.47) |
| Race/Ethnicity | Hispanic: 58 (52%)  NH, White: 43 (38%)  NH, NA/AN: 4 (4%)  NH, NH/PI: 2 (2%)  NH, Asian: 1 (1%)  NH, B/AA: 4 (4%) | Hispanic: 36 (49%)  NH, White: 30 (41%)  NH, NA/AN: 3 (4%)  NH, NH/PI: 0 (0%)  NH, Asian: 1 (1%)  NH, B/AA: 4 (5%) | Hispanic: 22 (58%)  NH, White: 13 (34%)  NH, NA/AN: 1 (3%)  NH, NH/PI: 2 (5%)  NH, Asian: 0 (0%)  NH, B/AA: 0 (0%) | 7.28 (0.20) |
| Highest Level of Education Completed | ≤ 8th Grade: 2 (2%)  Some HS: 18 (16%)  HS or equivalent: 42 (38%)  Some college/2-year degree: 41 (37%)  College graduate/4-year degree: 4 (4%)  Graduate/professional degree: 5 (4%) | ≤ 8th Grade: 2 (2%)  Some HS: 14 (19%)  HS or equivalent: 28 (38%)  Some college/2-year degree: 29 (39%)  College graduate/4-year degree: 0 (0%)  Graduate/professional degree: 1 (1%) | ≤ 8th Grade: 0 (0%)  Some HS: 4 (11%)  HS or equivalent: 14 (37%)  Some college/2-year degree: 12 (32%)  College graduate/4-year degree: 4 (11%)  Graduate/professional degree: 4 (11%) | 15.05 (0.01) |
| Insurance Status | Private: 11 (10%)  Public or None: 98 (88%)  Missing: 3 (3%) | Private: 3 (4%)  Public or None: 70 (95%)  Missing: 1 (1%) | Private: 8 (21%)  Public or None: 28 (74%)  Missing: 2 (5%) | 10.07, (<0.01) |

NA/AN: Native American or Alaskan Native; NH/PI: Native Hawaiian or Pacific Islander; B/AA: Black or African American; NH: Non-Hispanic; HS: High School

^1^ Test statistic value listed is chi-square or t value.
