# Supplementary material for: Compliance and Satisfaction With a Protocol for Identifying Novel Targets to Support Postpartum Opioid Use Disorder Recovery: Prospective Cohort Study
Source: JMIR Form Res. 2025 Nov 20;9:e77899. doi: 10.2196/77899 (PMC12633836; doi:10.2196/77899)
Supplement: Multimedia Appendix 6 [file formative-v9-e77899-s006.docx]

**Supplementary Document 6. Description of Study Sample – Completed at least 1 Postpartum Visit (n=61)**

|  | **Total**  **(n=61)** | **OUD+ (n=43)** | **OUD- (n=18)** | **Test Statistic**^1^ **(p-value)** |
| --- | --- | --- | --- | --- |
| **Sociodemographic Variables** | | | | |
| Age^2^ | 29.4±5.1 | 30.1±5.0 | 27.8±5.2 | 1.63 (0.11) |
| Race/Ethnicity | Hispanic: 31 (51%)  NH, White: 25 (41%)  NH, NA/AN: 3 (5%)  NH, NH/PI: 0 (0%)  NH, Asian: 1 (2%)  NH B/AA: 1 (2%) | Hispanic: 21 (49%)  NH, White: 18 (42%)  NH, NA/AN: 2 (5%)  NH, NH/PI: 0 (0%)  NH, Asian: 1 (2%)  NH B/AA: 1 (2%) | Hispanic: 10 (56%)  NH, White: 7 (39%)  NH, NA/AN: 1 (6%)  NH, NH/PI: 0 (0%)  NH, Asian: 0 (0%)  NH B/AA: 0 (0%) | 1.00 (0.91) |
| Highest Level of Education Completed | ≤ 8th Grade: 2 (3%)  Some HS: 8 (13%)  HS or equivalent: 22 (36%)  Some college/2-year degree: 25 (41%)  College graduate/4-year degree: 2 (3%)  Graduate/professional degree: 2 (3%) | ≤ 8th Grade: 2 (4%)  Some HS: 6 (14%)  HS or equivalent: 17 (39%)  Some college/2-year degree: 17 (39%)  College graduate/4-year degree: 0 (0%)  Graduate/professional degree: 1 (2%) | ≤ 8th Grade: 0 (0%)  Some HS: 2 (11%)  HS or equivalent: 5 (28%)  Some college/2-year degree: 8 (44%)  College graduate/4-year degree: 2 (11%)  Graduate/professional degree: 1 (6%) | 6.66 (0.25) |
| Insurance Status | Private: 5 (8%)  Public or None: 56 (92%)  Missing: 0 (0%) | Private: 1 (2%)  Public or None: 42 (97%)  Missing: 0 (0%) | Private: 4 (22%)  Public or None: 14 (78%)  Missing: 0 (0%) | 4.29 (0.04) |
| Number of Children Living in Home^2^ | 1.2±1.5 | 1.2±1.7 | 1.0±0.8 | 0.71 (0.48) |
| Gestational Week Prenatal Care Initiated^2^ | 9.0±5.1 | 9.7±5.4 | 7.3±3.9 | 1.92 (0.06) |
| Parity | Primiparous: 17 (28%)  Multiparous:44 (72%) | Primiparous: 11 (25%)  Multiparous: 32 (74%) | Primiparous: 6 (33%)  Multiparous: 12 (67%) | 0.09 (0.76) |
| Gestational Week at Baseline Visit^2^ | 36.8±0.8 | 36.8±0.8 | 36.8±0.6 | 0.02 (0.98) |
| Gestational Week at time of delivery^2^ | 39.5±1.0 | 39.6±1.0 | 39.2±0.8 | 1.73 (0.09) |
| Mother/Infant Discharged from Hospital at Same Time | 43 (70%) | 27 (63%) | 16 (89%) | 2.99 (0.08) |
| **Substance Use History** | | | | |
| Lifetime History of Use of Substances with Abuse Potential^3^ | Opioid-Containing Prescription: 51 (84%)  Heroin: 26 (43%)  Narcotics: 41 (67%)  Fentanyl: 5 (8%)  ----  Caffeine: 59 (97%)  Alcohol: 53 (87%)  Cigarettes/Nicotine: 50 (82%)  Cannabis: 48 (79%)  Cocaine: 37 (61%) | Opioid-Containing Prescription: 42 (98%)  Heroin: 26 (60%)  Narcotics: 41 (95%)  Fentanyl: 5 (12%)  ----  Caffeine: 41 (95%)  Alcohol: 38 (88%)  Cigarettes/Nicotine: 39 (91%)  Cannabis: 39 (91%)  Cocaine: 36 (84%) | Opioid-Containing Prescription: 9 (50%)  Heroin: 0 (0%)  Narcotics: 0 (0%)  Fentanyl: 0 (0%)  ---  Caffeine: 18 (100%)  Alcohol: 15 (83%)  Cigarettes/Nicotine: 11 (61%)  Cannabis: 9 (50%)  Cocaine: 1 (6%) | n/a |
| Use in 3 Months Prior to Pregnancy of Substances with Abuse Potential^3^ | Opioid-Containing Prescription: 13 (21%)  Heroin: 1 (2%)  Narcotics: 25 (41%)  Fentanyl: 4 (7%)  ---  Caffeine: 57 (93%)  Alcohol: 24 (39%)  Cigarettes/Nicotine: 38 (62%)  Cannabis: 31 (51%)  Cocaine: 4 (7%) | Opioid-Containing Prescription: 13 (30%)  Heroin: 1 (2%)  Narcotics: 25 (58%)  Fentanyl: 4 (9%)  ---  Caffeine: 39 (91%)  Alcohol: 10 (23%)  Cigarettes/Nicotine: 30 (70%)  Cannabis: 26 (60%)  Cocaine: 4 (9%) | Opioid-Containing Prescription: 0 (0%)  Heroin: 0 (0%)  Narcotics: 0 (0%)  Fentanyl: 0 (0%)  ---  Caffeine: 18 (100%)  Alcohol: 14 (78%)  Cigarettes/Nicotine: 8 (44%)  Cannabis: 5 (28%)  Cocaine: 0 (0%) | n/a |
|  | Opioid-Containing Prescription: 7 (11%)  Heroin: 0 (0%)  Narcotics: 35 (57%)  Fentanyl: 1 (2%)  ---  Caffeine: 54 (89%)  Alcohol: 2 (3%)  Cigarettes/Nicotine: 28 (46%)  Cannabis: 13 (21%)  Cocaine: 1 (2%) | Opioid-Containing Prescription: 7 (16%)  Heroin: 0 (0%)  Narcotics: 35 (81%)  Fentanyl: 1 (2%)  ---  Caffeine: 37 (86%)  Alcohol: 2 (5%)  Cigarettes/Nicotine: 25 (58%)  Cannabis: 13 (30%)  Cocaine: 1 (2%) | Opioid-Containing Prescription: 0 (0%)  Heroin: 0 (0%)  Narcotics: 0 (0%)  Fentanyl: 0 (0%)  ---  Caffeine: 17 (94%)  Alcohol: 0 (0%)  Cigarettes/Nicotine: 3 (17%)  Cannabis: 0 (0%)  Cocaine: 0 (0%) | n/a |
| Drug of choice (Top five) | - | Opioid-Containing Prescription: 16 (37%)  Heroin: 11 (26%)  Fentanyl: 6 (14%)  Cannabis: 3 (7%)  Caffeine: 3 (7%) | - | n/a |
| Age of first opioid-containing prescription use^2^ | - | 17.5±5.9  (n=41) | - | n/a |
| Age of first heroin use^2^ | - | 22.6±5.6  (n=26) | - | n/a |
| Lifetime History Treatment Type | - | Inpatient: 27 (63%)  Outpatient 26 (61%)  Intensive Outpatient: 23 (54%) | - | n/a |
| Current Treatment Type | - | Inpatient: 2 (5%)  Outpatient 25 (58%)  Intensive Outpatient: 5 (12%) | - | n/a |
| Current Treatment Components | - | Medication: 34 (79%) Counseling/Support Groups: 23 (54%)  Other: 2 (5%) | - | n/a |
| Length of Recovery | - | > 1 year: 19 (44%)  Before this pregnancy but <1 year: 8 (19%)  1^st^ Trimester of this pregnancy: 5 (12%)  2^nd^ Trimester of this pregnancy: 8 (19%)  3^rd^ Trimester of this pregnancy: 3 (7%) | - | n/a |

NA/AN: Native American or Alaskan Native; NH/PI: Native Hawaiian or Pacific Islander; B/AA: Black or African American; NH: Non-Hispanic; HS: High School

^1^ Test statistic value listed is chi-square or t value.

^2^ Values are mean ± standard deviation.

^3^ Substances listed include opioids and the five most commonly endorsed substances used per lifetime history.
