# Supplementary material for: Compliance and Satisfaction With a Protocol for Identifying Novel Targets to Support Postpartum Opioid Use Disorder Recovery: Prospective Cohort Study
Source: JMIR Form Res. 2025 Nov 20;9:e77899. doi: 10.2196/77899 (PMC12633836; doi:10.2196/77899)
Supplement: Multimedia Appendix 7 [file formative-v9-e77899-s007.docx]

**Supplementary Document 7. Protocol Completion by Time Point, Modality, and Group**

|  | **Total**  **(n=70)** | **OUD+ (n=50)** | **OUD- (n=20)** | **Test Statistic,  p-value** |
| --- | --- | --- | --- | --- |
| **Baseline Visit Completed** | **70 (100%)** | **50 (100%)** | **20 (100%)** | **-** |
| Interviews (During Visit) | 70 (100%) | 50 (100%) | 20 (100%) | - |
| Dried Blood Spots (During Visit) | 58 (83%) | 39 (78%) | 19 (95%) | 1.83, .17 |
| Saliva Samples (During Visit) | 53 (76%) | 34 (68%) | 19 (95%) | 4.29, .04 |
| Saliva Samples (Outside Visit) | 98 (70%) | 62 (62%) | 36 (90%) | 9.37, <.01 |
| Daily Surveys (Outside Visit) * | 370 (87%) | 250 (83%) | 120 (97%) | 13.9, <.01 |
| Weekly Surveys (Outside Visit) | 630 (100%) | 450 (100%) | 180 (100%) | - |
| **Week 1 Visit Completed** | **63 (90%)** | **45 (90%)** | **18 (90%)** | **0.00, >.99** |
| Interviews (During Visit) | 54 (77%) | 36 (72%) | 18 (90%) | 1.70, .19 |
| Dried Blood Spots (During Visit) | 49 (70%) | 33 (66%) | 16 (80%) | 0.75, .39 |
| Saliva Samples (During Visit) | 49 (70%) | 31 (62%) | 18 (90%) | 4.08, .04 |
| Saliva Samples (Outside Visit) | 94 (67%) | 59 (59%) | 35 (88%) | 9.27, <.01 |
| Daily Surveys (Outside Visit) | 337 (69%) | 222 (63%) | 115 (82%) | 15.45, <.01 |
| Weekly Surveys (Outside Visit) | 514 (82%) | 352 (78%) | 162 (90%) | 11.10, <.01 |
| **Week 2 Visit Completed** | **61 (87%)** | **43 (86%)** | **18 (90%)** | **0.03, .95** |
| Interviews (During Visit) | 51 (73%) | 33 (66%) | 18 (90%) | 3.03, .08 |
| Dried Blood Spots (During Visit) | 48 (69%) | 32 (64%) | 16 (80%) | 1.03, .31 |
| Saliva Samples (During Visit) | 45 (64%) | 28 (56%) | 17 (85%) | 4.05, .04 |
| Saliva Samples (Outside Visit) | 95 (68%) | 64 (64%) | 31 (78%) | 1.81, .18 |
| Daily Surveys (Outside Visit) | 350 (71%) | 233 (67%) | 117 (84%) | 13.34, <.01 |
| Weekly Surveys (Outside Visit) | 514 (82%) | 353 (78%) | 161 (89%) | 9.64, <.01 |
| **Week 3 Visit Completed** | **60 (86%)** | **42 (84%)** | **18 (90%)** | **0.07, .79** |
| Interviews (During Visit) | 52 (74%) | 35 (70%) | 17 (85%) | 0.99, .32 |
| Dried Blood Spots (During Visit) | 48 (69%) | 32 (64%) | 16 (80%) | 1.03, .31 |
| Saliva Samples (During Visit) | 49 (70%) | 32 (64%) | 17 (85%) | 2.08, .15 |
| Saliva Samples (Outside Visit) | 98 (70%) | 64 (64%) | 34 (85%) | 5.04, .02 |
| Daily Surveys (Outside Visit) | 320 (65%) | 207 (59%) | 113 (81%) | 19.60, <.01 |
| Weekly Surveys (Outside Visit) | 503 (80%) | 350 (78%) | 153 (85%) | 3.73, .05 |
| **Week 4 Visit Completed** | **58 (83%)** | **40 (80%)** | **18 (90%)** | **0.42, .51** |
| Interviews (During Visit) | 54 (77%) | 37 (74%) | 17 (85%) | 0.45, .50 |
| Dried Blood Spots (During Visit) | 47 (67%) | 31 (62%) | 16 (80%) | 1.36, .24 |
| Saliva Samples (During Visit) | 50 (71%) | 33 (66%) | 17 (85%) | 1.68, .19 |
| Saliva Samples (Outside Visit) | 100 (71%) | 66 (66%) | 34 (85%) | 4.16, .04 |
| Daily Surveys (Outside Visit) | 331 (68%) | 215 (61%) | 116 (83%) | 20.00, <.01 |
| Weekly Surveys (Outside Visit) | 818 (73%) | 563 (70%) | 255 (80%) | 9.60, <.01 |
| **Week 5 Visit Completed** | **58 (83%)** | **40 (80%)** | **18 (90%)** | **0.42, .51** |
| Interviews (During Visit) | 49 (70%) | 33 (66%) | 16 (80%) | 0.75, .39 |
| Dried Blood Spots (During Visit) | 49 (70%) | 34 (68%) | 15 (75%) | 0.08, .77 |
| Saliva Samples (During Visit) | 46 (66%) | 30 (60%) | 16 (80%) | 1.73, .19 |
| Saliva Samples (Outside Visit) | 95 (68%) | 61 (61%) | 34 (85%) | 6.48, .01 |
| Daily Surveys (Outside Visit) | 324 (66%) | 210 (60%) | 114 (81%) | 19.55, <.01 |
| Weekly Surveys (Outside Visit) | 474 (75%) | 322 (72%) | 152 (84%) | 10.78, <.01 |
| **Week 6 Visit Completed** | **58 (83%)** | **40 (80%)** | **18 (90%)** | **0.42, .51** |
| Interviews (During Visit) | 52 (74%) | 34 (68%) | 18 (90%) | 2.56, .11 |
| Dried Blood Spots (During Visit) | 49 (70%) | 34 (68%) | 15 (75%) | 0.08, .77 |
| Saliva Samples (During Visit) | 48 (69%) | 31 (62%) | 17 (85%) | 2.52, .11 |
| Saliva Samples (Outside Visit) | 95 (68%) | 61 (61%) | 34 (85%) | 6.48, .01 |
| Daily Surveys (Outside Visit) | 311 (63%) | 199 (57%) | 112 (80%) | 22.11, <.01 |
| Weekly Surveys (Outside Visit) | 477 (76%) | 318 (71%) | 159 (88%) | 20.87, <.01 |
| **Week 7 Visit Completed** | **57 (81%)** | **39 (78%)** | **18 (90%)** | **0.17, .67** |
| Interviews (During Visit) | 47 (67%) | 30 (60%) | 17 (85%) | 2.99, .08 |
| Dried Blood Spots (During Visit) | 41 (59%) | 27 (54%) | 14 (70%) | 0.92, .34 |
| Saliva Samples (During Visit) | 47 (67%) | 30 (60%) | 17 (85%) | 2.99, .08 |
| Saliva Samples (Outside Visit) | 90 (64%) | 56 (56%) | 34 (85%) | 9.24, <.01 |
| Daily Surveys (Outside Visit) | 301 (61%) | 193 (55%) | 108 (77%) | 19.51, <.01 |
| Weekly Surveys (Outside Visit) | 470 (75%) | 318 (71%) | 152 (84%) | 12.16, <.01 |
| **Week 8 Visit Completed** | **56 (80%)** | **38 (76%)** | **18 (90%)** | **0.98, .32** |
| Interviews (During Visit) | 52 (74%) | 34 (68%) | 18 (90%) | 2.56, .11 |
| Dried Blood Spots (During Visit) | 43 (61%) | 31 (62%) | 12 (60%) | 0.00, >0.99 |
| Saliva Samples (During Visit) | 48 (69%) | 31 (62%) | 17 (85%) | 2.52, .11 |
| Saliva Samples (Outside Visit) | 94 (67%) | 60 (60%) | 34 (85%) | 7.00, <.01 |
| Daily Surveys (Outside Visit) | 315 (64%) | 201 (57%) | 114 (81%) | 24.05, <.01 |
| Weekly Surveys (Outside Visit) | 779 (70%) | 512 (64%) | 267 (83%) | 25.74, <.01 |
| **Week 9 Visit Completed** | **55 (78%)** | **37 (74%)** | **18 (90%)** | **1.32, .25** |
| Interviews (During Visit) | 49 (70%) | 31 (62%) | 18 (90%) | 4.08, .04 |
| Dried Blood Spots (During Visit) | 40 (57%) | 26 (52%) | 14 (70%) | 1.23, .27 |
| Saliva Samples (During Visit) | 44 (63%) | 28 (56%) | 16 (80%) | 2.57, .11 |
| Saliva Samples (Outside Visit) | 90 (64%) | 58 (58%) | 32 (80%) | 5.10, .02 |
| Daily Surveys (Outside Visit) | 304 (62%) | 194 (55%) | 110 (79%) | 21.77, <.01 |
| Weekly Surveys (Outside Visit) | 464 (74%) | 303 (67%) | 161 (89%) | 31.26, <.01 |
| **Week 10 Visit Completed** | **55 (78%)** | **37 (74%)** | **18 (90%)** | **1.32, .25** |
| Interviews (During Visit) | 50 (71%) | 32 (64%) | 18 (90%) | 3.54, .06 |
| Dried Blood Spots (During Visit) | 38 (54%) | 27 (54%) | 11 (55%) | 0.00, >0.99 |
| Saliva Samples (During Visit) | 42 (60%) | 25 (50%) | 17 (85%) | 5.90, .01 |
| Saliva Samples (Outside Visit) | 80 (57%) | 46 (46%) | 34 (85%) | 16.19, <.01 |
| Daily Surveys (Outside Visit) | 304 (62%) | 196 (56%) | 108 (77%) | 18.09, <.01 |
| Weekly Surveys (Outside Visit) | 465 (74%) | 303 (67%) | 162 (90%) | 33.00, <.01 |
| **Week 11 Visit Completed** | **54 (77%)** | **36 (72%)** | **18 (90%)** | **1.70, .19** |
| Interviews (During Visit) | 49 (70%) | 31 (62%) | 18 (90%) | 4.08, .04 |
| Dried Blood Spots (During Visit) | 43 (61%) | 29 (58%) | 14 (70%) | 0.43,.51 |
| Saliva Samples (During Visit) | 44 (63%) | 28 (56%) | 16 (80%) | 2.57, .11 |
| Saliva Samples (Outside Visit) | 88 (63%) | 55 (55%) | 33 (82%) | 8.11, <.01 |
| Daily Surveys (Outside Visit) | 301 (61%) | 194 (55%) | 107 (76%) | 17.74, <.01 |
| Weekly Surveys (Outside Visit) | 468 (74%) | 307 (68%) | 161 (89%) | 29.21, <.01 |
| **Week 12 Visit Completed** | **54 (77%)** | **36 (72%)** | **18 (90%)** | **1.70, .19** |
| Interviews (During Visit) | 50 (71%) | 32 (64%) | 18 (90%) | 3.54, .06 |
| Dried Blood Spots (During Visit) | 37 (53%) | 25 (50%) | 12 (60%) | 0.24, .62 |
| Saliva Samples (During Visit) | 43 (61%) | 26 (52%) | 17 (85%) | 5.25, .02 |
| Saliva Samples (Outside Visit) | 87 (62%) | 53 (53%) | 34 (85%) | 11.11, <.01 |
| Daily Surveys (Outside Visit) | 300 (61%) | 189 (54%) | 111 (79%) | 25.88, <.01 |
| Weekly Surveys (Outside Visit) | 766 (68%) | 496 (62%) | 270 (84%) | 26.89 <.01 |
| **Month 4 Visit Completed** | **52 (74%)** | **35 (70%)** | **17 (85%)** | **0.99, .32** |
| Interviews (During Visit) | 50 (71%) | 33 (66%) | 17 (85%) | 1.68, .19 |
| Dried Blood Spots (During Visit) | 42 (60%) | 26 (52%) | 16 (80%) | 3.57, .06 |
| Saliva Samples (During Visit) | 46 (66%) | 28 (58%) | 17 (85%) | 4.06, .04 |
| Saliva Samples (Outside Visit) | 88 (63%) | 56 (56%) | 32 (80%) | 6.06, .01 |
| Daily Surveys (Outside Visit) | 323 (66%) | 213 (61%) | 110 (79%) | 13.19, <.01 |
| Weekly Surveys (Outside Visit) | 766 (68%) | 511 (64%) | 255 (80%) | 8.43, <.01 |
| **Month 5 Visit Completed** | **51 (73%)** | **34 (68%)** | **17 (85%)** | **1.32, .25** |
| Interviews (During Visit) | 46 (66%) | 30 (60%) | 16 (80%) | 1.73, .19 |
| Dried Blood Spots (During Visit) | 34 (49%) | 21 (42%) | 13 (65%) | 2.17, .14 |
| Saliva Samples (During Visit) | 43 (61%) | 27 (54%) | 16 (80%) | 3.05, .08 |
| Saliva Samples (Outside Visit) | 88 (63%) | 56 (56%) | 32 (80%) | 6.06, .01 |
| Daily Surveys (Outside Visit) | 280 (57%) | 169 (48%) | 111 (79%) | 37.98, <.01 |
| Weekly Surveys (Outside Visit) | 743 (66%) | 488 (61%) | 255 (80%) | 34.91, <.01 |

* During the 7 days preceding birth.
